# A Time-Space Domain Information Fusion Method for Specific Emitter Identification Based on Dempster–Shafer Evidence Theory

**DOI:** 10.3390/s17091972

**Published:** 2017-08-28

**Authors:** Wen Jiang, Ying Cao, Lin Yang, Zichang He

**Affiliations:** 1 School of Electronics and Information, Northwestern Polytechnical University, Xi’an 710072, China; caoyingnwpu@hotmail.com (Y.C.); hezichang@mail.nwpu.edu.cn (Z.H.); 2 China Equipment System Engineering Company, Beijing 100039, China

**Keywords:** Dempster–Shafer evidence theory, specific emitter identification, time–space domain information fusion, quantum mechanical approach, correlation coefficient, recursive centralized model

## Abstract

Specific emitter identification plays an important role in contemporary military affairs. However, most of the existing specific emitter identification methods haven’t taken into account the processing of uncertain information. Therefore, this paper proposes a time–space domain information fusion method based on Dempster–Shafer evidence theory, which has the ability to deal with uncertain information in the process of specific emitter identification. In this paper, radars will generate a group of evidence respectively based on the information they obtained, and our main task is to fuse the multiple groups of evidence to get a reasonable result. Within the framework of recursive centralized fusion model, the proposed method incorporates a correlation coefficient, which measures the relevance between evidence and a quantum mechanical approach, which is based on the parameters of radar itself. The simulation results of an illustrative example demonstrate that the proposed method can effectively deal with uncertain information and get a reasonable recognition result.

## 1. Introduction

Information fusion, also known as multi-sensor data fusion , is a process of synthesizing data or information obtained from multiple sources to achieve a certain purpose, which has been widely used in many fields, such as detection [1,2,3], recognition [4,5], tracking [6,7,8,9], image processing [10,11,12], fault diagnosis [13,14,15,16,17], and gender profiling [18]. In recent years, the rapid development of sensor technology and computer technology has greatly promoted the research of information fusion technology [19,20,21,22]. As an uncertain information processing method that satisfies the weaker conditions than traditional Bayesian probability theory, Dempster–Shafer evidence theory [23,24] is widely adopted by scholars in information fusion [25,26,27,28,29].

Information fusion incorporates space-domain fusion and time-domain fusion. In space domain, it is a process of fusing multiple sets of evidence generated by multiple sensors in space. Since the credibility of a body of evidence generated by a single sensor can not be determined, the integration of multi-sensor information in space-domain is beneficial to people in order to make more accurate judgements than a single sensor. However, it doesn’t take into account the changes in evidence over time. While in practical applications, the information obtained by different measurement periods may change due to the interference of other factors, and at this time, the time-domain fusion, which is a fusion process of evidence generated at different moments with the properties of dynamic, sequential and real-time, is very important. In general, the time-domain and the space-domain are both indispensable in the process of the information fusion. At present, there are a variety of space-domain information fusion results [30,31,32,33,34,35,36], and the time domain information fusion has also been gradually adopted by scholars [37,38,39]. Hong and Lynch [40] proposed the space-time information fusion models based on evidence theory, including recursive centralized fusion model, recursive distribution without feedback fusion model and recursive distribution with feedback fusion model. In addition, Hong [41] made profound analysis and comparison of these three models. However, they didn’t give these three models specific application background. In this paper, the recursive centralized model is applied to specific emitter identification.

Radar plays an important role in contemporary military affairs. However, the modern electromagnetic environment is complex since there are signals from a number of different emitters present, and signals from the same emitter are noisy or their parameters (features) are not measured with a great accuracy [42]. Therefore, how to distinguish the correct information from the acquired signal and obtain a reasonable result is a difficult problem. This field is called radar target recognition (RTR), which is widely used in command automation, identification friend or foe and intelligence acquisition and meets the needs of modern high-tech warfare and helps to realize the intelligence and informatization of radar. In RTR, there is a process of source (target) recognition and classification and identification. Recognition concerns type classification, while identification focuses recognition on particular copies of the same radar (target) type [43]. The identification process is more specialized as it requires methods based on the analysis of distinctive features. These features are then identified based on the information obtained. This is called specific emitter identification (SEI). The main task of SEI is to identify radar emission sources. Although there is much research about SEI [43,44,45], most of them do not consider the processing of uncertain information, which means that the identification method in the references [43,44,45] start from the point of view of signal processing. However, Dempster–Shafer evidence theory holds that we can only say radar is more likely to belong to which type, not to say which radar definitely belongs to which type. Therefore, in this paper, we assume that radar will give a set of evidence about the specific identity of the unknown target after signal analysis. Our main task is to fuse multiple sets of evidence to identify the identity of an unknown target.

He and Jiang [46] proposed a quantum mechanical approach based on Dempster–Shafer evidence theory, which considered work performance of radars themselves to model reliability of sensor reports, while most of the previous methods only considered the interrelationships between evidence generated by sensors. However, the quantum mechanical approach [46] failed to deal with the impact of time factors on the fusion results effectively. Therefore, in this paper, a time–space domain information fusion method for SEI based on Dempster–Shafer evidence theory is proposed. At first, as the distance between the radar and the target will change over time, the fusion of time-domain evidence is joined. Then, the proposed method adopts correlation coefficient [47] and quantum mechanical approach to generate weighting factors (weights). At last, within the framework of recursive centralized model, the evidence is combined based on Dempster’s combination rule. The space-time domain information fusion method of SEI proposed in this paper takes full account of the influence of time factors on the fusion results so that it has a strong dynamic nature. In addition, it incorporates the interrelationship between evidence that is measured by correlation coefficient and the impact of the radar’s own performance, which considered by a quantum mechanical approach at the same time, so its management of conflict information is more comprehensive.

The structure of this paper is as follows: in Section 2, the relevant background knowledge is introduced, including Dempster–Shafer evidence theory, correlation coefficient and recursive centralized fusion model. Section 3 describes the quantum mechanical approach and discuss it. Section 4 introduces the specific steps of the proposed method. In addition, the proposed method is simulated in Section 5. Section 6 presents the conclusions.

## 2. Preliminaries

### 2.1. Dempster–Shafer Evidence Theory

Dempster–Shafer evidence theory (D–S evidence theory) [23,24] is more extensive than traditional Bayesian probability with it satisfying the weaker prior conditions, and this concept contributes to its good performance in handling the uncertainty information [48,49,50]. It is briefly introduced as follows.

**Definition** **1.** *Suppose* Θ *is a nonempty finite set composed of n exhaustive and exclusive elements, and it satisfies:*

(1) $$\Theta =\{\theta_{1},\theta_{2},\cdots \theta_{i},\cdots ,\theta_{N}\},$$

*where set* Θ *is called a frame of discernment. The power set of* Θ, $2^{\Theta }$, *is indicated as:*

(2) $$2^{\Theta }=\left\{\emptyset ,\left\{\theta_{1}\right\},\cdots \left\{\theta_{N}\right\},\left\{\theta_{1},\theta_{2}\right\},\cdots ,\left\{\theta_{1},\theta_{2},\cdots \theta_{i}\right\},\cdots ,\Theta \right\}.$$

**Definition** **2.** *A mass function is a mapping m from $2^{\Theta }$ to [0,1], formally defined as:*

(3) $$m:2^{\Theta }\to \left[0,1\right],$$

*and it satisfies:*

(4) m(∅)=0,

(5) $$\sum_{A\in 2^{\Theta }}m(A)=1.$$

The function *m* is called Basic Probability Assignment (BPA), and m(A) indicates the degree of trust in proposition A. If m(A)>0, A is called a focal element of Θ. If m(A)=0, it means that the proposition is totally lacks belief. In addition, a value between [0,1] indicates partial belief.

**Definition** **3.** *In D–S theory, the combination is denoted as* ⊕. *Supposing that there are two BPAs denoted by $m_{1}$ and $m_{2}$, the two BPAs’ combination with the Dempster’s combination rule is then formulated as follows:*

(6) $$m_{⊕}\left(A\right)=\frac{\sum_{B\cap C=A}m_{1}\left(B\right)m_{2}\left(C\right)}{1-k},\;A\neq \emptyset ,$$

(7) $$m_{⊕}\left(\emptyset \right)=0,$$

*where*

(8) $$k=m\left(\emptyset \right)=\sum_{B\cap C=\emptyset }m_{1}\left(B\right)m_{2}\left(C\right).$$

Essentially, *k* reflects the degree of conflict among evidence. In D–S theory, if k=0, we say that the two evidence is fully compatible with each other. On the contrary, if k=1, we say that the two pieces of evidence are in total conflict with each other. In addition, the Dempster’s combination rule is commutative and associative, which ensures that the fusion result has nothing to do with the order of the fusion process, and it essentially assigns the mass of empty set to each set by the use of the normalization. It has two characteristics:

- Pieces of mutual support evidence are reinforced.
- Pieces of conflict evidence weaken each other.

However, the direct use of the Dempster’s combination rule to integrate the evidence generated by information sources may produce counterintuitive results [51].

### 2.2. Correlation Coefficient of Belief Function

How to measure conflict between evidence is still an open issue. In classical D–S evidence theory, the mass of empty set *k* is used to reflect the degree of conflict among evidence. Actually, *k* defines the compatibility between the evidence. Jousselme [52] proposed evidence distance to measure conflict from the perspective of the similarity between evidence. Liu [53] proposed a two-dimensional measure that contains k and evidence distance and considers the compatibility and similarity at the same time. In addition, many other scholars studied the problem [54,55,56,57]. In this section, we will introduce a new correlation coefficient [47], which measures the relevance between evidence and contains compatibility and similarity based on our previous work.

**Definition** **4.** *A discernment frame is assumed with n elements, and two pieces of evidence are denoted by $m_{1}$ and $m_{2}$. The correlation coefficient is defined as Equation (9):*

(9) $$r_{BPA}\left(m_{1},m_{2}\right)=\frac{c(m_{1},m_{2})}{\sqrt{c\left(m_{1},m_{1}\right)\cdot c\left(m_{2},m_{2}\right)}},$$

*where*

(10) $$c\left(m_{1},m_{2}\right)=\sum_{i=1}^{2^{n}}\sum_{j=1}^{2^{n}}m_{1}\left(A_{i}\right)m_{2}(A_{j})\frac{|A_{i}\cap A_{j}|}{|A_{i}\cup A_{j}|},$$

*where $i,j=1,2,\cdots ,2^{n}$; $A_{i}$ and $A_{j}$ are the elements of BPAs $m_{1}$ and $m_{2}$; In addition, |·| is the cardinality of a subset. Actually, $c(m_{1},m_{2})$ shows the degree of correlation.*

The correlation coefficient $r_{BPA}$ measures the relevance between evidence $m_{1}$ and $m_{2}$. The larger the $r_{BPA}$, the greater the relevance between the evidence, and the lower the degree of conflict. In extreme cases, we can say that evidence is in total conflict if $r_{BPA}=0$ and in the total absence of conflict if $r_{BPA}=1$.

### 2.3. Recursive Centralized Model

Hong and Lynch [40] studied time–space information fusion model based on D–S evidence theory. Until now, there are three basic models: recursive centralized fusion model, recursive distribution without feedback fusion model and recursive distribution with feedback fusion model [40]. The recursive centralized fusion model that is used in our method can realize real-time fusion and high precision of data processing.

**Figure 1:**
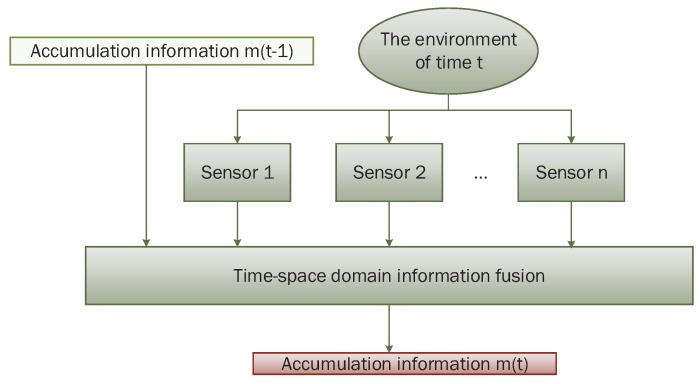
Recursive centralized model.

The recursive centralized fusion process is shown in Figure 1. At moment *t*, it first fuses N evidence generated by N sensors, and then fuses it with cumulative information denoted as m(t−1). The fused result could include total target identification information at the moment t.

### 2.4. Radar Working Principle

**Figure 2:**
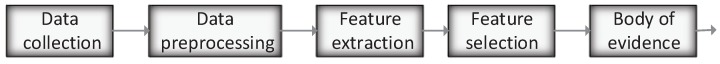
Working process of radar.

Radar is a device that detects and measures information of targets by transmitting electromagnetic waves and receiving echoes. The specific use and structure of the various radars are different, but the basic form is consistent, including: transmitter, transmitting antenna, receiver, receiving antenna, processing part and display. In addition, there is also power equipment, data entry equipment, anti-jamming equipment and other auxiliary equipment. Generally speaking, radars are divided into active radars and passive radars. Their main difference is that the active radar transmits and receives signals, while the passive radar only receives signals. In this paper, we adopt passive radar. The process of radar information processing is shown in Figure 2. In this paper, radar generates a piece of evidence based on the information obtained that include: relative position, speed, heading, closest point of approach (CPA) and distance to CPA.

In general, parameters related to the performance of the radar are shown in Definitions 5 and Equation (12).

**Definition** **5.** *Since the passive radar only receives the signal, the signal is one-way. The one-way signal strength received by radar is defined as follows:*

(11) $$P_{r}=\frac{P_{t}G_{t}G_{r}\sigma \lambda^{2}}{{\left(4\pi x\right)}^{2}},$$

*where $P_{t}$ defines the transmission ability of radar, with $G_{t}$ the gain of target antenna, $G_{r}$ the gain of radar antenna, σ the radar cross section (including the geometric cross-sectional area, reflection coefficient and direction coefficient) factor, r the wavelength, and x the distance between the target object and the radar.*

**Definition** **6.** *The maximal reconnaissance distance is defined as follows:*

(12) $$x_{r}={\left[\frac{P_{t}G_{t}G_{r}\sigma \lambda^{2}}{{\left(4\pi \right)}^{2}P_{r\min}}\right]}^{\frac{1}{2}},$$

*where $P_{r\min}$ is the minimum signal strength radar can receive.*

If the distance between radar and target beyond $x_{r}$, the signal strength received by radar is too small for radar to detect it properly.

## 3. Existing Quantum Mechanical Approach

He and Jiang [46] proposed a quantum mechanical method that considers the performance of the radar sensor itself to assign weights among evidence based on the link of uncertainty between quantum mechanics and D–S evidence theory. In this section, we will introduce this method and discuss it.

**Figure 3:**
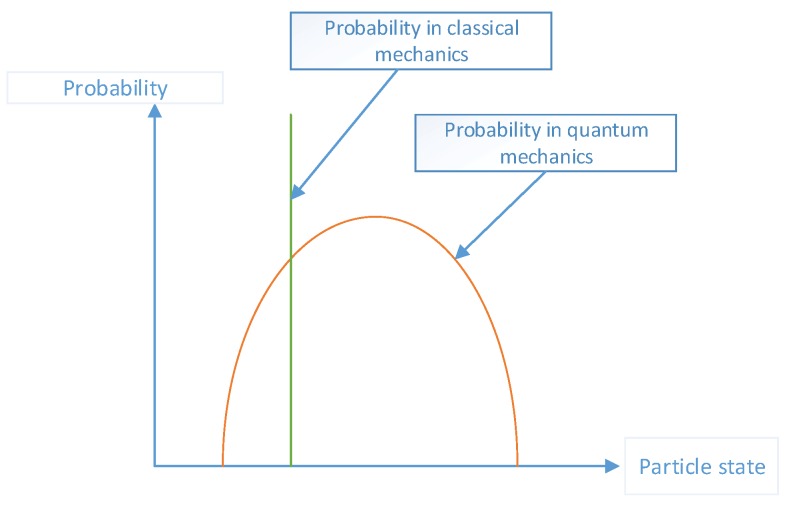
Particle state and probability.

In classical mechanics, it is generally believed that state of a particle is determined at some points in time where the state of the particle is usually expressed by the coordinates and momentum of the particle. However, the position and momentum of the particle can not be determined at the same time according to the Heisenberg uncertainty principle in quantum mechanics, and they can only be in the form of probability. Therefore, we can only say that particles are more likely to be in which state, but can not be sure the particles is in one state as Figure 3 shows.

In classical mechanics, if the state of the particle is known at a given moment, the state of the particle can be obtained at any time after the particle is determined according to its equation of motion. Similarly, in quantum mechanics, when the state of the microscopic particles is known at some point, the state of motion of the particles can also be determined by an equation, which is called Schrodinger equation.

**Definition** **7.** *One state becomes stationary when the energy of a system in which the particle located is a certain value.The equation of the Schrodinger equation is as follows:*

(13) $$-\frac{\hbar^{2}}{2m}\nabla^{2}\Psi +U\left(\vec{r}\right)\Psi =E\Psi ,$$

*where E is total energy (also called the overall state) of the system,* Ψ *represents wave function, $-\frac{\hbar^{2}}{2m}$ is kinetic energy, $U(\vec{r})$ is Potential energy and* ∇ *is Laplacian operator. $-\frac{\hbar^{2}}{2m}\nabla^{2}+U\left(\vec{r}\right)$ is called Hamiltonian operator.*

The wave function depicts the quantum state of the system, which is proportional to the probability that the intensity of a point in the space (the square of the absolute value of the amplitude) and the probability of finding the particle at that point. According to this interpretation, the wave describing the particle is a probability wave. Actually, it is essentially a probability distribution curve that represents the probability that the particle is in the corresponding state. According to this property, Bolotin [58] solved the Schrodinger equation with a membership curve of the problem solution, and then the optimal solution of the problem is obtained. In Bolotin’s approach, the most important step is to set up quasi-potential function and then put it into the Schrodinger equation and solve it. Similarly, since the performance of the radar is related to signal-to-noise ratio (SNR), and SNR is related to the received signal strength, we regard the signal strength as quasi-potential function, shown in Equation (14):

(14) $$U\left(x\right)=\{\begin{array}{c} \frac{\gamma }{x^{2}}\;0<x<x_{r}\\ \infty \;x\leq 0,x>x_{r}, \end{array}$$

where

(15) $$\gamma =\frac{P_{t}G_{t}G_{r}\sigma \lambda^{2}}{{\left(4\pi \right)}^{2}}.$$

γ corresponds to the parameters of Equation (11). In addition, it is impossible for a particle to penetrate the well wall if it is within a infinite well potential, so Ψ(x)=0 when the distance beyond the maximal reconnaissance distance.

In addition, then, we put U(x) into the Schrodinger equation and solve it, we can get the wave function. In addition, with it, the probability distribution curve P(x) can be obtained in Equation (16):

(16) $$P\left(x\right)=|\Psi \left(x\right){|}^{2}\propto x{\left[J_{\alpha }\left(\frac{\sqrt{L}}{c}\right)+Y_{\alpha }\left(\frac{\sqrt{L}}{c}\right)\right]}^{2},$$

where $J_{\alpha }$ and $Y_{\alpha }$ are Bessel functions of the first kind and the second kind, respectively [59], and $\alpha =\frac{1}{2}\sqrt{\frac{c^{2}-4\gamma }{c^{2}}}$, which denotes their order.

**Figure 4:**
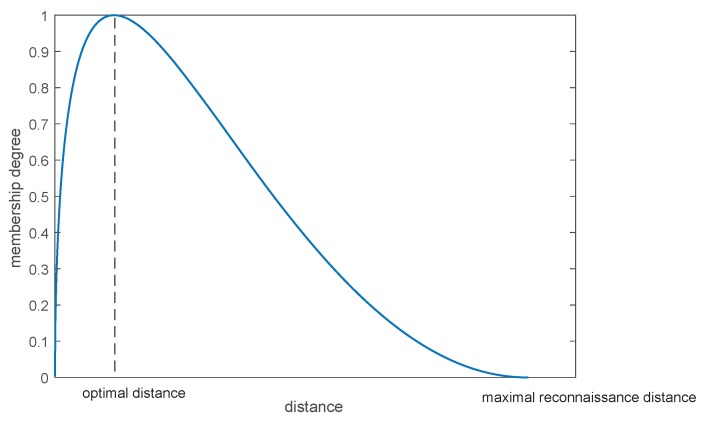
Radar’s working distance and membership degree.

**Figure 5:**
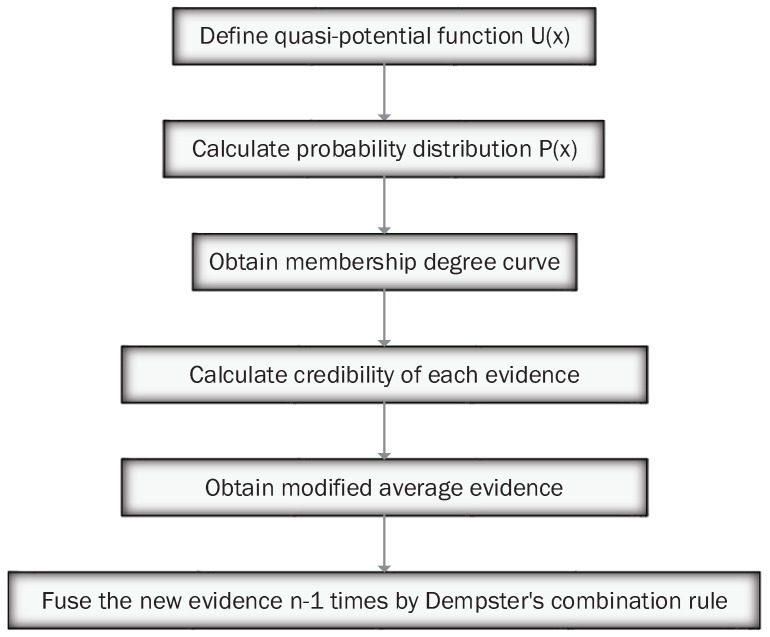
The process of the existing quantum mechanical approach.

Normalizing probability distribution, we can obtain the relationship curve between radar working distance and membership, shown in Figure 4. The higher the membership degree is when the radar is at a certain working distance, the better performance of the radar, and the more reliable the evidence generated by the radar is. Deng [30] proposed a method of the distribution of weights based on evidence distance that measures the similarity between evidence. Similarly, weights among evidence can be assigned based on the memberships we obtained that show the performance of multiple radars themselves. The process of the quantum mechanical approach is shown in Figure 5.

## 4. Proposed Method to Realize SEI

He and Jiang [46] opened the way to consider the performance of radar work, but their method failed to effectively deal with the impact of time on the entire process. In this section, a time–space domain information fusion method for SEI will be proposed, which considers both the relationship between evidence and performance of radars themselves based on evidence theory and the existing quantum mechanical approach.

Most of the current methods of information fusion are carried out in space-domain that is based on multi-sensor fusion. However, in real applications, the information obtained by a single measurement cycle has a certain contingency as it may be interfered with by electromagnetic waves, weather and other factors. In addition, this kind of contingency can be prevented by time-domain information fusion. In addition, the system of time-domain fusion has properties of real-time, sequence and dynamic, which can achieve inheritance and update of the integration. In this paper, the evidence and distance between radars and target will change over time. In addition, the confidence of evidence generated by radar sensors may then be different at different moments if we select multiple sets of evidence at multiple moments.

Now, supposing that there are *n* radars and *t* measurement cycles, the specific steps of the method are shown as follows:

Step 1 Calculate the correlation coefficient, and then get the weighting factors based on evidence. $r_{kj}$ is the correlation coefficient between evidence *k* and *j*, and then we can get a matrix:
  (17) $$R=\left[\begin{array}{cccc} 1 & r_{12} & \cdots & r_{1n}\\ r_{21} & 1 & \cdots & r_{2n}\\ \vdots & \vdots & \vdots & \vdots \\ r_{n1} & r_{n2} & \cdots & 1 \end{array}\right].$$
  The degree of trust of a piece of evidence can be defined as:
  (18) $$\lambda_{j}=\sum_{k=1,k\neq j}^{n}r_{kj}.$$
  However, since we are in the system of time-domain fusion where evidence is different at different moments, $\lambda_{j}$ is different at different moments. Therefore, it needs to be defined as $\lambda_{ij}$, which represents the support degree of radar *j* at moment *i*.
Step 2 Calculate membership degree curve of each radar by quantum mechanical approach based on the performance parameters of given radars, and then get the membership degree $\omega_{ij}$ of the corresponding working distance at each moment.
Step 3 Calculate the final weights $\mu_{ij}$, which is the final weight of a piece of evidence generated by radar *j* at moment *i* by summing and normalizing:
  (19) $$\mu_{ij}=\frac{\omega_{ij}\cdot \lambda_{ij}}{\sum_{j=1}^{n}\left(\omega_{ij}\cdot \lambda_{ij}\right)}.$$
Step 4 The credibility of each evidence $\mu_{ij}$ is regarded as a weighting factor to weight the evidence at moment *i*, and then we get a new piece of modified average evidence (MAE):
  (20) $$MAE=\sum_{j=1}^{n}\left(\mu_{ij}\cdot m_{i}\right).$$
Step 5 Get the fusion results of the current moment by fusing the new piece of evidence n−1 times, and then fuse the fusion results of the current moment and the previous moment by Dempster’s combination rule. This is based on the recursive centralized model.

**Figure 6:**
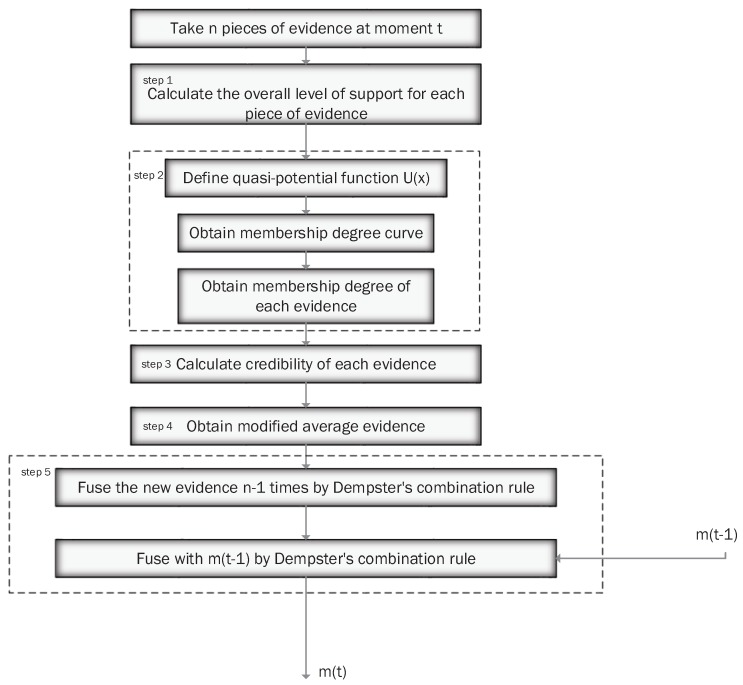
The process of the proposed method.

The process of the proposed method is shown in Figure 6. In this method, the parameter $\lambda_{ij}$ represents the weight that comes from the perspective of relationships between evidence. While $\omega_{ij}$ is the weight on the radar itself’s perspective. We find a balance denoted by $\mu_{ij}$ between these two kinds of weights when we multiply the two parameters. Therefore, the proposed method not only considers the data’s relationship, but also the performance of the sensor itself, while most of the existing methods only consider one issue. In the next section, the proposed method will be simulated and compared with other existing methods.

## 5. Case Study

In this section, we simulate the data fusion process of radar practically by fusing the assumed BPAs, and several simulation results are presented here to test the performance of the proposed method of SEI. Our analysis process is carried out from three aspects:

- Comparing the proposed method with Dempster, Deng et al. [30] and He and Jiang [46] when the target moves in a straight line.
- Comparing the proposed method with the other three methods when the target moves along a curve.
- Using self performance comparison when the number of radars changes and when we only sample one moment, the target moves in a straight line at five moments.

**Figure 7:**
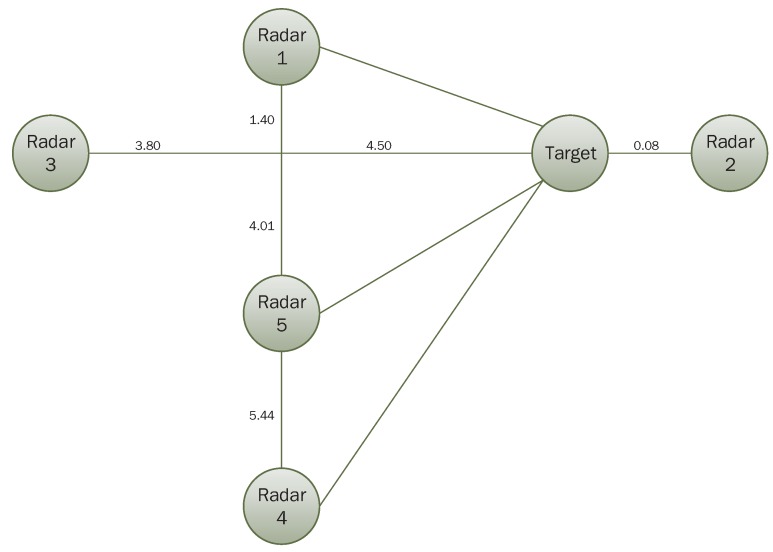
Relative position of five radars with the target when the target moves along a straight line.

Assume frame of discernment is: Θ={A,B,C}, which means that the identity of the unknown target is one of the three elements. In this section, we assume that “A” is the correct identification result. There are five radars distributed in space and they are likely to be disturbed in the region to be monitored because of weather, noise or some other factors. Their relative positions with the target are shown in Figure 7, and the target travels along a straight line to radar 3. Now, we select five moments denoted by $t_{1}$,$t_{2}$,$t_{3}$,$t_{4}$ and $t_{5}$, and the BPAs generated at different moments by the five radars based on information received are shown in Table 1.

**Figure 8:**
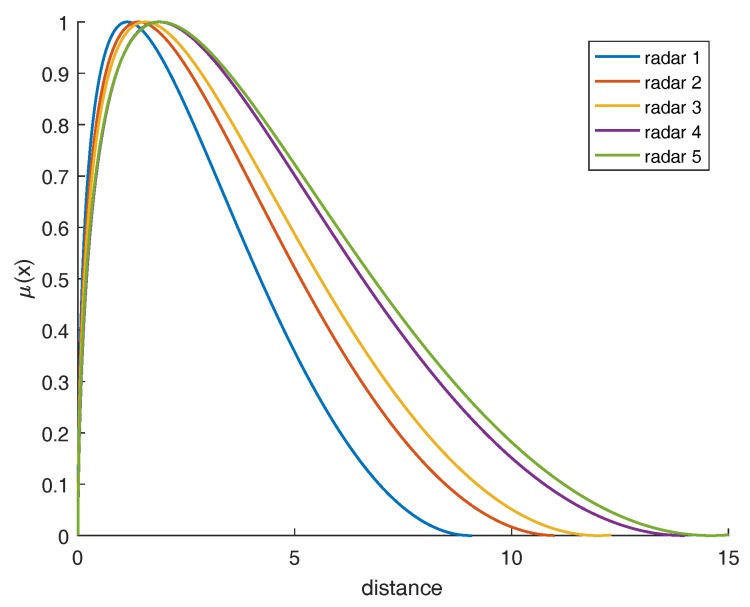
The relationship between distance and membership degree.

**Figure 9:**
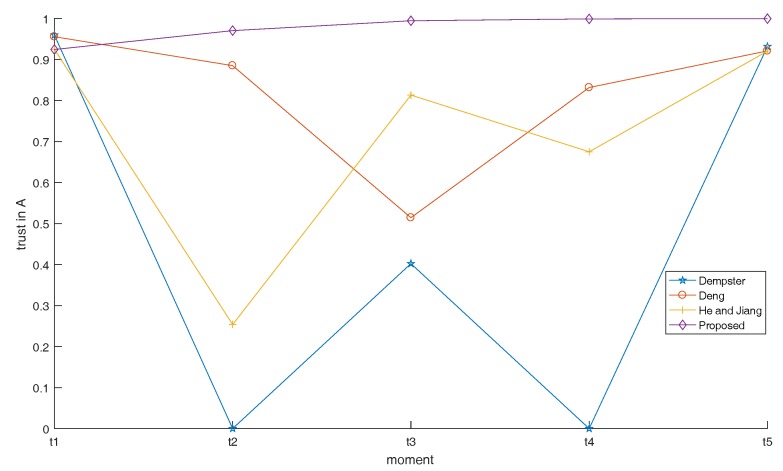
The trend of the results at five moments when the target moves along a straight line.

The maximum reconnaissance distances for five radars are 9.1, 11.0, 12.3, 14.0 and 15.1, and performance parameters *c*, *L* and γ are shown in Table 2, where *c* is used to control the width of the curve, *L* is the inherent state of the whole system, and it is proportional to $P_{r\min}$. γ is shown in Equation (15), representing the radar’s own parameters. Five curves can be obtained that describe the relationship between distance and membership degree (shown in Figure 8). The distance between radar and target has been changing over time, so the radar’s membership degree at different moments are different. Distances between five radars and target at five moments are shown in Table 3. According to the distances between the radar and the target, we can get the membership degree (that is, the performance of the five radars). In addition, the final results can then be calculated, which are shown in Table 4. The trend of the results at five moments is shown in Figure 9.

In the process of simulation above, the SEI method proposed in this paper was compared with three existing methods, including Dempster’s combination rule, Deng’s combination rule [30], and He and Jiang’s method [46]. Firstly, at moment $t_{1}$, all of the radars are working properly. In this case, all four of the methods yield reasonable results. Secondly, the classical Dempster’s combination rule has a characteristic named “one-vote veto”, which means that if one piece out of all of the evidence gives the proposition “A” complete negation, the final fusion result will completely negate “A” (like the time $t_{2}$, and $t_{4}$). We can see that the proposed method of this paper solved this problem. Thirdly, He and Jiang’s quantum mechanical approach [46] opened up the precedence of the distribution of weights based on the radar’s own performance. However, they didn’t take the impact of previous results on the current moment and relationship among evidence into account. In this case, it is likely to produce unreasonable results if a disturbed radar at one moment happens to work at the degree of membership of 1, but other radars’ memberships are small, such as the evidence generated at $t_{2}$. In addition, at this moment, interrelationship between evidence (correlation coefficient [47] applied in this paper) needs to be reconciled, and our method solves this problem. Finally, Deng [30]’s rule only considered the interrelationship among evidence and its strategy’s ability of anti-interference is poor. As in the case of $t_{3}$, when there are two radars that are interfered with and produce the wrong BPAs, Deng’s result will have a higher degree of uncertainty or even a false result. In this paper, we introduce time-domain fusion that takes the effect of the previous result at the current moment into account. In this case, even if the trust in “A” (the correct result) is not very high due to the interference in the current time, “A” will be still have a high degree of trust after recursive centralized fusion if the previous result supports “A”. This reflects the ability of anti-interference and the integration of the results of the succession and update.

**Figure 10:**
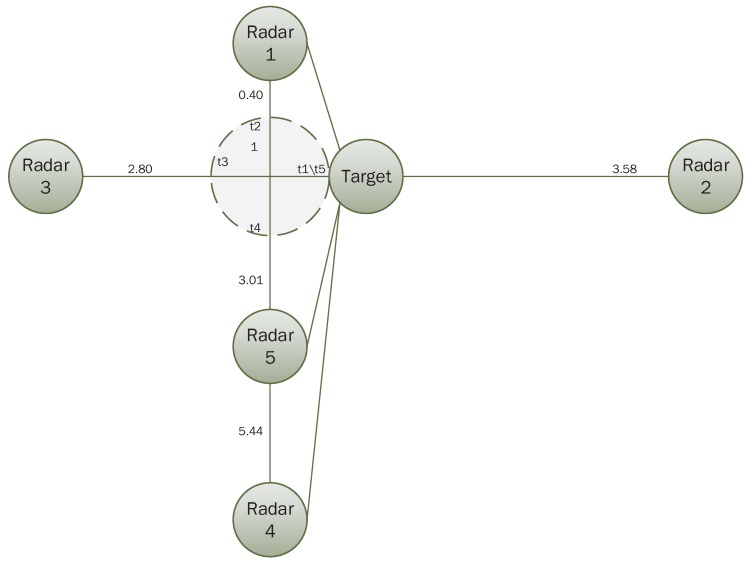
Relative position of five radars with the target when the target moves along curves.

**Figure 11:**
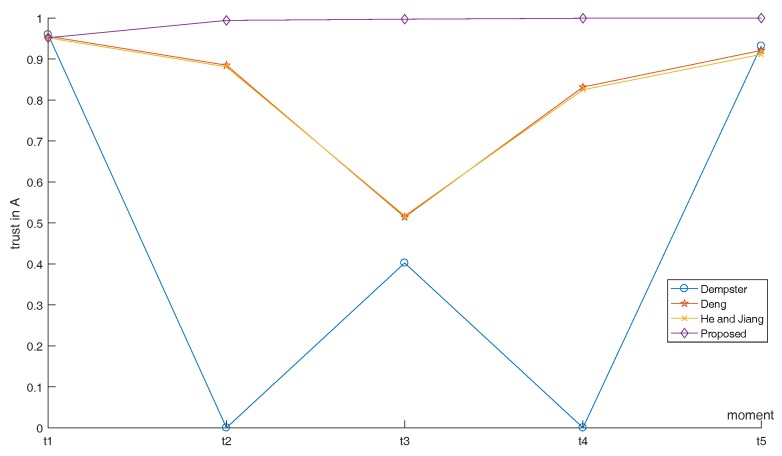
The trend of the results at five moments when the target moves along curves.

Now, we change the movement trajectory of the target, and then the relative position of five radars with the target changes with it. However, the performance parameters of the five radars and BPAs in five moments do not change. Therefore, we compare the proposed method with the other three methods. The simulation process is shown below. We assume that the target moves along a circular path with a radius of 1 as shown by the dotted line in Figure 10 and the spatial location of five radars is the same as with Figure 7. Starting from the moment $t_{1}$, the unknown target moves a quarter of the circle at every moment, and back to the origin at moment $t_{5}$. Distances between five radars and the unknown target at five moments are shown in Table 5. According to the distances between the radars and the target, we can get the membership degree with the basis of Figure 8. In addition, the final results of proposed method and the other three methods can then be calculated, which is shown in Table 6. The trend of the results at five moments is shown in Figure 11.

Since Dempster and Deng [30]’s combination results are only relevant to the data given to the evidence, but not to the relative position of the radar with the target, their combination results at five moments are the same as Table 4 if we change the movement trajectory of the target. When we change the movement trajectory of the target, the fusion results also change since the performance of He and Jiang’s method [46] has a great relationship with distance between target and radar. The proposed method is in the same situation. From Figure 11, we can see that the uncertainty of the fusion results of the proposed method is always low but fluctuates with He and Jiang’s method [46] in five moments. Why does this happen? As we discussed before, the three fusion methods that we adopted to compare with our method only consider the BPAs of the current moment. However, in practical applications, it is difficult for us to decide which moment to determine. The proposed method takes the effect of the previous fusion result into account. Therefore, in general, the robustness of the proposed method is better than the other three methods.

**Figure 12:**
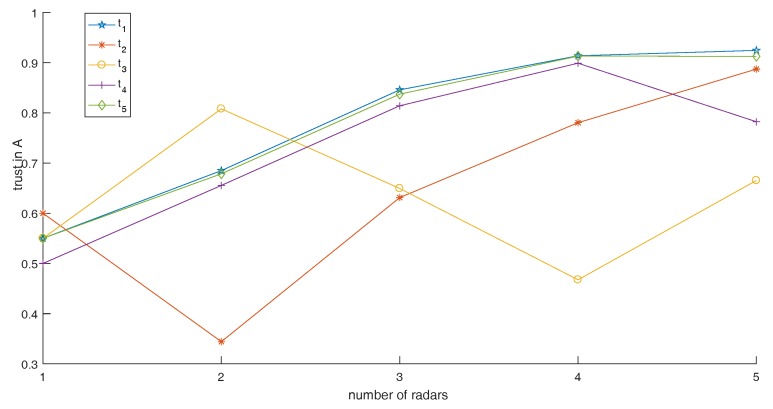
The trend of fusion results from different numbers of radars.

Now, we analyze the effect of variations in radar number on fusion results. BPAs of five radars at five moments do not change. From the value of BPAs, we can see that five radars are working properly since they all support the proposition “A” at $t_{1}$, while there are some radars not working properly at $t_{2}$, $t_{3}$, $t_{4}$ and $t_{5}$. Since the effect of variations in radar number on the performance of the proposed method in this simulation needed to be determined, we only use self performance comparison of the proposed method at five moments. In addition, the relative positions of the five radars with targets is the same as Figure 7. When a different number of radar sensors are used in our identification system, the fusion results are shown in Table 7. In Table 7, we assume that only the BPAs of the current time are considered in order to show the importance of sampling at multiple moments. The trend of fusion results from different numbers of radars are shown in Figure 12.

From Figure 12, we can say that the more radars that are in the identification system, the lower the uncertainty of the results are if all radars are working properly (as shown by the results of the moment $t_{1}$). Why does this happen? As mentioned in Section 2, Dempster’s combination rule, which is a part of our method, has a characteristic: mutual support evidence is reinforced. Therefore, the greater the number of radars support “A”, the greater the trust given to “A” in the fusion results. However, what will happen if some radars are not working properly? Dempster’s combination rule has another characteristic: pieces of conflict evidence weaken each other. Therefore, the uncertainty of the fusion results will rise if some radars are not working properly. For example, radar supports proposition “B”, so we get unreasonable results if we just use the first two radars. At moment $t_{3}$, radar 3 and radar 4 are not working properly, while moment $t_{4}$ is radar 5 and moment $t_{4}$ is also radar 5. From Figure 12, when we join the radars that are not working properly, trust given to “A” declines. Logically speaking, we can get more information if there are more radars in the identification system, and then fusion results obtained are more reliable. If the number of radars is too small, it is likely that the number of radars that are not working properly is almost the same as those working properly, even if the number of radars not working properly is higher. In addition, in this situation, there will be a high degree of uncertainty or even fusion results that are completely wrong.

This is also the case for the number of sampled moments. The more points that are sampled in the time domain, the more information is obtained. Looking at the data from the last column of Table 7, if we only sample at one moment, the fusion results are likely to have higher uncertainty. Therefore, in general, whether all the radars are working properly or some radars are not working properly, we should try to use a larger number of radars and more moments to get more information. It is obvious that the greater amount of information, the more favorable it is for us to get reasonable results.

## 6. Conclusions

Specific Emitter Identification technology is one of the key technologies of the modern electronic warfare system and electromagnetic environment monitor. Most of the previous methods have been proposed from the point of view of feature signal processing. In addition, they did not consider dealing with uncertain information. Therefore, in this paper, we started from the point of view of information fusion and proposed a new identification method based on D–S evidence theory, as it is a powerful tool to deal with uncertain information. At first, we simulated the actual radar data with assumed BPAs. Secondly, we changed the number of radars and movement trajectory, respectively, to simulate the effect of the number and relative position on the final results. From the simulation results, final results of the proposed method have low uncertainty and good robustness. In addition, the greater the number of radars and sampling points is, the more information we obtained, making the fusion results more reliable. Thirdly, from the calculation principle of this proposed method, it considers not only the relationship between the BPAs (evidence), but also the performance of radars themselves to generate weights. This is more comprehensive than the previous methods, as most of the previous methods only considered one of the two influencing factors. Fourthly, since the distance between radar and target will change over time, we join time-domain evidence fusion. Time-domain fusion can prevent unreasonable results that generate occasionally at a single moment, which misleads the final decision-making.

In summary, vertical strategy is applied firstly, that is, the fusion results are generated by assigning weights at a single moment and fusing the modified average evidence. We then use the horizontal strategy (time-domain recursive fusion) to demonstrate the inheritance and updating of the fusion results. Finally, the computational burden of the proposed method mainly exists in Dempster’s combination rule. In addition, the computational complexity of Dempster’s combination rule increases exponentially with the cardinality of the discernment frame. In this paper, computational burden is small as we only use BPAs of five radars at five moments to simulate the practical situation in MatLab (MATLAB R2016b, MathWorks, Natick, MA, USA). In real conditions, hardware acceleration can be used to reduce the computational burden. This is the next step we want to study. In addition, the spatial positions of the five radars in the simulation of this paper are man-made. In future work, we can consider designing a new algorithm from the perspective of signal processing to realize the optimal configuration of multiple radars, so as to generate reasonable BPAs and then make the identification results more reliable.

## Figures and Tables

**Table 1:** Basic Probability Assignments generated at different moments by the five radars.

		$m_{1}$	$m_{2}$	$m_{3}$	$m_{4}$	$m_{5}$
$t_{1}$	{A}	0.55	0.5	0.65	0.5	0.5
	{B}	0.25	0.35	0.2	0.2	0.15
	{C}	0.1	0.05	0.1	0.2	0.1
	{A,B,C}	0.1	0.1	0.05	0.1	0.25
$t_{2}$	{A}	0.6	0	0.5	0.5	0.55
	{B}	0.15	0.95	0.3	0.1	0.25
	{C}	0.2	0.05	0.1	0.15	0.1
	{A,B,C}	0.05	0	0.1	0.25	0.1
$t_{3}$	{A}	0.6	0.6	0	0.1	0.55
	{B}	0.1	0.2	0.6	0.7	0.35
	{C}	0.1	0.1	0.15	0.1	0.05
	{A,B,C}	0.2	0.1	0.25	0.1	0.05
$t_{4}$	{A}	0.55	0.4	0.55	0.5	0
	{B}	0.2	0.3	0.2	0.1	0.5
	{C}	0.1	0.2	0.2	0.2	0.5
	{A,B,C}	0.15	0.1	0.05	0.2	0
$t_{5}$	{A}	0.5	0.5	0.6	0.55	0.4
	{B}	0.2	0.2	0.2	0.35	0.4
	{C}	0.1	0.2	0.1	0.1	0.1
	{A,B,C}	0.2	0.1	0.1	0	0.1

**Table 2:** The value of performance parameters.

	*c*	*L*	γ
radara	16	0.64	47.25
radarb	16	0.41	48.13
radarc	16	0.31	49.75
radard	16	0.17	53.01
radare	16	0.20	50.49

**Table 3:** Distances between five radars and target at five moments when the target moves along a straight line.

	Radar1	Radar2	Radar3	Radar4	Radar5
$t_{1}$	4.7127	0.08	8.3	10.4667	6.0274
$t_{2}$	3.31	1.58	6.8	9.92	5.01
$t_{3}$	2.0447	3.08	5.3	9.5737	4.2837
$t_{4}$	1.4	4.58	3.8	9.45	4.01
$t_{5}$	2.0447	6.08	1.3	9.5737	4.2837

**Table 4:** Simulation results of four methods when the target moves along a straight line.

		$t_{1}$	$t_{2}$	$t_{3}$	$t_{4}$	$t_{5}$
$Dempster^{\prime }s\;rule$	{A}	0.9595	0	0.4022	0	0.9317
	{B}	0.0368	0.9790	0.5863	0.5863	0.0613
	{C}	0.0036	0.0210	0.0109	0.4167	0.0068
$Deng^{\prime }s\;rule$	{A}	0.9555	0.8849	0.5142	0.8316	0.9209
	{B}	0.0395	0.1055	0.4764	0.0972	0.0735
	{C}	0.0048	0.0093	0.0088	0.0710	0.0055
$He\;and\;Jiang^{\prime }s\;method$	{A}	0.9249	0.2538	0.8132	0.6749	0.9203
	{B}	0.0712	0.7435	0.1791	0.2087	0.0733
	{C}	0.0035	0.0026	0.0071	0.1163	0.0061
Proposedmethod	{A}	0.9243	0.9703	0.9944	0.9989	0.9995
	{B}	0.0718	0.0294	0.0055	0.0009	0.0002
	{C}	0.0036	0.0002	0.0001	0.0001	0.0002

**Table 5:** Distance between five radars and the target at five moments when the target moves along curves.

	Radar1	Radar2	Radar3	Radar4	Radar5
$t_{1}$	1.72	3.58	4.8	9.5	4.13
$t_{2}$	0.4	4.69	3.93	10.45	5.01
$t_{3}$	1.72	5.58	2.8	9.5	4.13
$t_{4}$	2.4	4.68	3.93	8.45	3.01
$t_{5}$	1.72	3.58	4.8	9.5	4.13

**Table 6:** Simulation results when the target moves along curves.

		$t_{1}$	$t_{2}$	$t_{3}$	$t_{4}$	$t_{5}$
$Dempster^{\prime }s\;rule$	{A}	0.9595	0	0.4022	0	0.9317
	{B}	0.0368	0.9790	0.5863	0.5863	0.0613
	{C}	0.0036	0.0210	0.0109	0.4167	0.0068
$Deng^{\prime }s\;rule$	{A}	0.9555	0.8849	0.5142	0.8316	0.9209
	{B}	0.0395	0.1055	0.4764	0.0972	0.0735
	{C}	0.0048	0.0093	0.0088	0.0710	0.0055
$He\;and\;Jiang^{\prime }s\;method$	{A}	0.9516	0.8808	0.5571	8429	0.9112
	{B}	0.0446	0.1099	0.4732	0.0914	0.0799
	{C}	0.0036	0.0091	0.0085	0.0654	0.0086
Proposedmethod	{A}	0.9518	0.9942	0.9972	09995	0.9999
	{B}	0.0444	0.0055	0.0026	0.0004	0.00005
	{C}	0.0033	0.0003	0.0002	0.0001	0.00005

**Table 7:** Simulation results of the proposed method when the number of radars changes.

NumberofRadars		1	2	3	4	5
$t_{1}$	{A}	0.55	0.6849	0.8457	0.9136	0.9243
	{B}	0.25	0.2586	0.1388	0.0807	0.0718
	{C}	0.1	0.0387	0.0135	0.0054	0.0036
$t_{2}$	{A}	0.6	0.3442	0.6311	0.7803	0.8871
	{B}	0.1	0.5897	0.3354	0.2024	0.1073
	{C}	0.1	0.0641	0.0324	0.0171	0.0055
$t_{3}$	{A}	0.55	0.8079	0.6494	0.4675	0.6653
	{B}	0.2	0.0883	0.2668	0.4789	0.3235
	{C}	0.1	0.0629	0.0632	0.0457	0.0103
$t_{4}$	{A}	0.5	0.6554	0.8140	0.8988	0.7822
	{B}	0.2	0.2144	0.1215	0.0652	0.1384
	{C}	0.1	0.1001	0.0607	0.0350	0.0792
$t_{5}$	{A}	0.55	0.6785	0.8371	0.9129	0.9125
	{B}	0.25	0.1711	0.1048	0.0648	0.0786
	{C}	0.1	0.1097	0.0501	0.0209	0.0086
